# Fatal Brucellosis Infection in a Liver Transplant Patient: A Case Report and Review of the Literature

**DOI:** 10.1155/2021/1519288

**Published:** 2021-06-18

**Authors:** Daniela Piedrahita, Alvaro Jose Martinez-Valencia, Olga Lucia Agudelo Rojas, Eric Tafur, Fernanto Rosso

**Affiliations:** ^1^Facultad de Ciencias de la Salud, Universidad Icesi, Cali, Colombia; ^2^Department of Internal Medicine, Infectious Disease Service, Fundación Valle del Lili, Cali, Valle del Cauca, Colombia; ^3^Fundación Valle del Lili, Centro de Investigaciones Clínicas (CIC), Cali, Valle del Cauca, Colombia

## Abstract

Brucellosis is the most common zoonosis, particularly in developing countries. The true incidence of human brucellosis is unknown. The WHO points out that 500,000 cases of brucellosis are reported each year from around the world. In Colombia, there is currently no regular surveillance of the event in humans and its prevalence is low due a low clinical suspicion. We report a case of a 66-year-old man, an urban merchant, who had received a liver transplant 11 years ago. The patient presented to the emergency department for two months of fatigue, severe myalgia, paresis of the extremities, loss of muscle strength, and progressive deterioration of functional class. In the emergency room, he became disoriented and was transferred to the intensive-care unit. He had a white blood cell count of 18990/uL and creatine phosphokinase 10302 U/L. Routine blood cultures were positive for *Brucella melitensis*. The patient reported consumption of unpasteurized bovine milk. He was treated with doxycycline and ciprofloxacin. Despite antibiotic management, after one month of hospitalization and in the context of septic shock with multiorgan failure, the patient died. Brucellosis is an unsuspected and underdiagnosed disease. It can occur in people with or without risk factors. Although the mortality is low, immunocompromised patients can develop fatal infections. A presumptive diagnosis can be established through the correlation of patient history and classic laboratory findings, which include transaminitis, anemia, and leukopenia with relative lymphocytosis; however, other findings can help us to guide the diagnosis, such as rhabdomyolysis, which appears as a complication in different infections; however, it had not been described before in brucellosis. A partnership between clinical suspicion laboratory diagnostic tests and improved disease surveillance systems is necessary to fight the disease.

## 1. Background

Brucellosis is the most common zoonotic disease worldwide, predominating in developing countries. It is caused by *Brucella* species, an intracellular, Gram-negative coccobacillary bacteria [1]. Humans acquire the disease by ingestion of unpasteurized dairy or inhalation or direct contact with secretions of infected animals such as cattle, goats, sheep, and pigs [2]. Farmers, butchers, and veterinarians are at high risk of infection [2].

Because in transplants patients, opportunistic infections such as zoonosis have shown an ascending trend due to the immunosuppression, brucellosis is being increasingly recognized after solid organ transplantation [3]. However, at present, insufficient evidence is accessible on the prevalence and treatment of brucellosis and the result of brucellosis in transplant population [4]. Brucellosis in solid organ transplantation (SOT) recipients has been reported, mainly in kidney transplant recipients. The time from transplantation to the infection is variable. The sources of infections in SOT recipients can be before and after transplantation, some of them including donor-derived infections, blood transfusion, reactivation of old infection, and new infections [5]. In transplant patients, this infection has a variable incubation period (days to months) and can present acutely, in a chronic form, as subclinical or asymptomatic, or as a severe systemic infection posing a real diagnostic challenge to the physician [6]. Initially, there can beundulant fever, diaphoresis, malaise, and weight loss; however, osteoarticular involvement is the most frequent focal complication [7]. Also, the neurologic, ganglionary, cardiac, genitourinary, renal, pulmonary, hepatobiliary, and multisystemic compromise can be present [8]. Four of the six defined *Brucella* species in animals are pathogenic to humans. *Brucella melitensis* is the most common and the most virulent bacterium that causes human disease [9]. The infection caused by *Brucella melitensis* is characterized by chronic and recurrent infections [10]. The diagnosis depends mainly on a high suspicion index. The cultures of blood, secretions, and tissues are the “gold standard.” However, the sensitivity of them varies from 15–70%. The Castañeda and lysis centrifugation blood culture techniques have been classically used due to a high rate of positive results within a time of incubation of 4 weeks [11]. Nowadays, automated, radiometric culture systems can recover the bacteria with high sensitivity and in lower time (3 days). The time of incubation is prolonged from 5 to 10 days. *Brucella*-specific serological tests may be helpful in prolonged disease, where cultures may have lower sensitivity. Nucleic acid amplification tests are another diagnosis alternative; however, it is not widely available [12]. The treatment is complex because it is an intracellular infection. Monotherapy is prohibited due to a high rate of relapses. It is recommended to initiate doxycycline plus streptomycin or rifampicin for 6 weeks. If neither of the last two can be used, the alternatives are gentamicin, ciprofloxacin, or trimethoprim-sulfamethoxazole (TMP-SMZ) [13]. In these patients, treatment usually involves longer courses of antibiotics. It is important to consider that drug interactions and organ dysfunction may limit antimicrobial agent options. Therefore, the management of each patient must be individualized. However, despite adequate treatment, some patients with the severe compromised immune system may have fatal outcomes or recurrent disease [6]. We report a case of a patient with a hepatic transplant 11 years ago who presented with brucellosis, after consuming unpasteurized bovine milk. This patient developed nonspecific symptoms for two months, which progressively worsened, and despite proper antibiotic therapy, he died.

## 2. Case Presentation

A 63-year-old man, a merchant from the urban area of Cali, Colombia, who had received a hepatic transplant 11 years ago, was admitted to the emergency department because of severe myalgia, paresis of extremities, loss of muscle strength, and progressive deterioration of functional class with gradual limitation of daily activities, permanent fatigue, and mild dyspnea. He also referred since two weeks ago the appearance of jaundice and choluria.

The patient had a history of arterial hypertension, type 2 diabetes mellitus, hypothyroidism, hyperlipidemia, and obesity. He received hepatic transplantation from a deceased donor 11 years ago after he had a diagnosis with end-stage liver disease due to nonalcoholic fatty liver disease. Medications included cyclosporine and mycophenolate with poor adherence.

On examination, the patient was disorientated and somnolent. He was transferred to the intensive-care unit. The temperature was 36.3°C, blood pressure 150/100 mmHg, pulse 84 beats/min, and respiratory rate 18 breaths/min. White blood cell count was 18990/uL (with 86,5% neutrophils and 5,20% lymphocytes), hemoglobin 16,9 g/dL, platelet count 268000/uL, and C-reactive protein (PCR) 0.64 (normal, 127 0–0.5 mg/dL). The serum level of aspartate aminotransferase was 999 U/L, alanine aminotransferase 511 U/L, alkaline phosphatase 53 U/L, and creatine phosphokinase 10302 U/L. Ultrasonography of the abdomen, eco-Doppler, and cholangioresonance were normal. The contrasted magnetic brain resonance showed nonspecific hyperintensity in the frontal subcortical white matter, and the lumbar puncture was normal. A multifactorial delirium was considered. Statin myopathy and graft rejection were suspected in the beginning; however, the hepatic biopsy was negative for graft rejection. In the search for infectious causes, cultures were taken. The urine and blood cultures were positive for *K. pneumoniae and E. coli*, both sensitive to ceftriaxone. Two days later, the microbiology laboratory informed that the blood cultures were also positive for *Brucella melitensis.* After identification of *B. melitensis* as the causative organism, doxycycline and gentamicin were added to manage, and rifampicin was contraindicated due to the severe hepatic dysfunction. The course was complicated by acute renal failure (creatine 4.15 mg/dL), requiring hemodialysis, and gentamicin was stopped. Ciprofloxacin was added in change. He developed respiratory failure, and he required mechanical ventilation and vasoactive support. The antimicrobial therapy escalated to linezolid, meropenem, doxycycline, ciprofloxacin, and caspofungin. New cultures were negative. He developed ascites causing him compression of the inguinal canal and scrotal edema. He was not in a condition for surgery. After a month of hospitalization and in the context of septic shock with multiorgan failure, the patient died.

## 3. Discussion

We report a case of a patient with a hepatic transplant 11 years ago who presented with brucellosis due to consuming unpasteurized bovine milk. This patient developed nonspecific symptoms for two months, which progressively worsened, and despite proper antibiotic therapy, the patient developed progressive septic shock with multiorgan failure and died.

In Colombia, brucellosis is not a notifiable disease and most of the seroprevalence data are from high-risk populations such as farmers, butchers, and veterinarians. This poses a limitation in the reasoning of clinicians who initially do not consider the disease and, therefore, do not perform a precise interrogation of risk factors. In our case, the positive blood culture findings allowed the identification of previous consumption of unpasteurized bovine milk during reinterrogation.

Because human brucellosis can affect any organ and body system, the presenting symptoms of the infection are not pathognomonic, and therefore, the disease may be easily confused with other medical conditions [1]. The differential diagnosis includes infectious and noninfectious diseases that share clinical characteristics with brucellosis such as typhoid fever, malaria, tuberculosis, dengue, yellow fever, rheumatoid arthritis, systemic lupus erythematosus, and lymphoma. Transplant recipients may present graft rejection, which is usually one of the first diagnoses to be ruled out at admission [9].

Hepatic complications are expected in brucellosis, and so, it is, thus, not surprising that it can react by ascites formation even in previously healthy patients or in the context of generalized fulminant disease course in patients with chronic liver involvement [9].

The symptoms and signs of brucellosis are highly variable, especially in immunosuppressed populations [1]. Previously reported cases of brucellosis in solid organ transplants showed a tendency to have more severe disease, usually with the involvement of multiple organs. We found three previously reported cases of brucellosis in liver transplant recipients.  Table 1 describes the course of the disease in previous reports. In these cases, the age and time after transplantation were highly variable. Despite immunosuppression, fever was the most frequent symptom in all cases. The diagnosis in several cases reviewed was made by blood culture; however, other techniques such as a serology and bone marrow culture, agglutination test, and immunocapture test were also used. In our case, routine automated blood culture allowed us to make a diagnosis on the second day; despite the fact that when the patient presented to our hospital, the disease probably had two months of duration.

Treatment of brucellosis is widely available in our setting; however, in patients with multiple comorbidities, it may be limited. In our case, the presence of renal and hepatic failure limited the antibiotic therapy. The patient was treated with doxycycline and ciprofloxacin. Rifampicin and gentamicin were not used because of liver and renal dysfunction. In brucellosis, it is known that the age of the patient and his comorbidities relate to the outcome. According to the literature, the average duration of the disease before diagnosis and treatment in adults is 40 days; however, in patients with osteoarticular diseases, it can be longer [6]. It is recommended that if there is a high suspicion of brucellosis, the physician should order a longer culture incubation that may range from 5 to 10 days. Brucella-specific serodiagnostic assays and nucleic acid amplification tests may be helpful.

It is imperative to generate preventive strategies involving consumers and health-care workers, to disclose the importance of suspecting this type of disease, since it may be present in people with no risk factors for acquiring it, and in those who have underlying conditions, that may lead to fatal outcomes. A partnership between clinical suspicion, better laboratory diagnostic tests, and improved disease surveillance systems is necessary to fight the disease.

## 4. Conclusions

Brucellosis is an unsuspected and underdiagnosed disease. A high suspicion index in addition to better diagnosis tests could improve clinical outcomes. However, despite adequate treatment, prolonged evolution time and a compromised immune system can lead to a fatal outcome.

## Figures and Tables

**Table 1:** Previous reported cases of brucellosis in liver transplant recipients.

Case	1	2	3	4
Author/year of publication	POLAT/2012	ISLEK/2013	XIE/2014	ROSSO/2021
Age in years/gender/country	15/M/Turkey	7/F/Turkey	39/M/China	63/M/Colombia
Time after transplant	2 months	2 years	2 years	10 years
Clinical features	Fever, diarrhea, headache, and herpetic lesion	Fever	Fever, sweats, cough, headache, and insomnia	Fatigue, dispnea, myalgia, paresis of the extremities, and jaundice
Diagnosis	Serology and bone marrow culture	Agglutination test and immunocapture test	Blood culture	Blood culture
Complication	Hematologic	None	None	Brain, lung, kidney, and spleen abscesses
Treatment	Doxycycline and rifampin for 8 weeks	TMP-SMZ and rifampicin for 12 weeks	TMP-SMZ and rifampicin for 8 weeks	Doxycycline and ciprofloxacin
Outcome	Alive	Alive	Alive	Death

## Data Availability

The datasets used to support the findings of this study are available from the corresponding author upon request.

## Abbreviations

WHO: World Health Organization
SOT: Solid organ transplantation.
